# Retraction Note: Deciphering the role of Hashimoto’s Thyroiditis-related key genes in thyroid cancer via detailed in silico analysis followed by the experimental validation

**DOI:** 10.1186/s41065-026-00754-y

**Published:** 2026-10-13

**Authors:** Mostafa A. Abdel-Maksoud, Taghreed N. Almana, Saeedah Almutair, Abdulaziz Alamri, Ibrahim A. Saleh, Mohamed Y. Zaky, Wahidah H. Al-Qahtani, Yasir Hameed

**Affiliations:** 1 Department of Botany and Microbiology, College of Science, King Saud University, P.O. Box 2455, Riyadh, 11451 Saudi Arabia; 2 Biochemistry Department-College of Science-King, Saud University, Riyadh, Saudi Arabia; 3 Faculty of Science, Zarqa University, Zarqa, 13110 Jordan; 4 Division of Hematology and Oncology, Department of Medicine, UPMC Hillman Cancer Center, University of Pittsburgh, Pittsburgh, PA 15213 USA; 5 Department of Food Sciences & Nutrition, College of Food and Agricultural Sciences, King Saud University, P.O. Box 270677, Riyadh, 11352 Saudi Arabia; 6 Department of Biochemistry and Biotechnology, The Islamia University of Bahawalpur, Bahawalpur, Pakistan

**Retraction Note: Hereditas 162, 91 (2025)**

**https://doi.org/10.1186/s41065-025-00429-0**

The Editor-in-Chief has retracted this article. After publication, concerns were raised regarding the in vitro images presented in Fig. 12 of this article, where the patterns of cells and colonies do not appear consistent with natural cell growth and proliferation. It has also been noted that a number of references in this article do not appear to support the statements preceding the citations.

The Editor-in-Chief therefore no longer has confidence in the presented data.

Dr. Yasir Hameed has stated on behalf of all authors that they disagree with this retraction.

